# Mutational analysis of βCOP (Sec26p) identifies an appendage domain critical for function

**DOI:** 10.1186/1471-2121-9-3

**Published:** 2008-01-22

**Authors:** Carol J DeRegis, Peter B Rahl, Gregory R Hoffman, Richard A Cerione, Ruth N Collins

**Affiliations:** 1 Graduate Program in Comparative Biomedical Sciences, Cornell University, Ithaca NY 14853, USA; 2 Graduate Program in Pharmacology, Cornell University, Ithaca, NY 14853, USA; 3 Graduate Program in Biophysics, Cornell University, Ithaca, NY 14853, USA; 4 Department of Molecular Medicine, Cornell University, Ithaca, NY 14853, USA; 5 Department of Chemistry and Chemical Biology, Cornell University, Ithaca, NY 14853, USA; 6 Department of Molecular Medicine, Cornell University, C4-109 Veterinary Medical Center, Ithaca, NY 14853, USA

## Abstract

**Background:**

The appendage domain of the γCOP subunit of the COPI vesicle coat bears a striking structural resemblance to adaptin-family appendages despite limited primary sequence homology. Both the γCOP appendage domain and an equivalent region on βCOP contain the FxxxW motif; the conservation of this motif suggested the existence of a functional appendage domain in βCOP.

**Results:**

Sequence comparisons in combination with structural prediction tools show that the fold of the COOH-terminus of Sec26p is strongly predicted to closely mimic that of adaptin-family appendages. Deletion of the appendage domain of Sec26p results in inviability in yeast, over-expression of the deletion construct is dominant negative and mutagenesis of this region identifies residues critical for function. The ArfGAP Glo3p was identified via suppression screening as a potential downstream modulator of Sec26p in a manner that is independent of the GAP activity of Glo3p but requires the presence of the COOH-terminal ISS motifs.

**Conclusion:**

Together, these results indicate an essential function for the predicted βCOP appendage and suggest that both COPI appendages perform a biologically active regulatory role with a structure related to adaptin-family appendage domains.

## Background

The intracellular transport vesicles of eukaryotic cells are responsible for ferrying cellular cargoes between membrane-bound compartments of the secretory and endocytic systems. Many of these vesicles are formed by a protein coat which functions both to select and trap cargo and to deform the membrane as part of the budding process. COPI-coated vesicles are responsible for the retrograde transport of cargo from the cis-Golgi back to the ER and have a role in intra-Golgi transport (reviewed in [1-4]). The COPI coat consists of seven coatomer subunits which are conserved across eukaryotes [5-7]. With high salt treatment the oligomer dissociates into [8]: (i) the F-COPI sub-complex, which resembles the adaptin heterotetramers and is composed of β-, γ-, δ-, and ζCOP (Sec26p/109 kDa, Sec21p/105 kDa, Ret2p/61 kDa, and Ret3p/22 kDa in *S. cerevisiae*, respectively), and (ii) the B-COPI sub-complex, composed of α-, β'-, and εCOP (Sec33p/136 kDa, Sec27p/99 kDa, and Sec28p/34 kDa in *S. cerevisiae*, respectively) with homology to clathrin [9]. The genes encoding each of these proteins are essential for cell survival in yeast, with the exception of *SEC28 *(εCOP) [10].

F-COPI and adaptin tetramers such as AP2 are composed of two large subunits (α and β2 in AP2), a medium subunit (μ2 in AP2), and a small subunit (σ2 in AP2). The structure of the AP2 holocomplex reveals two large subunits composed of helical trunk domains at the NH_2_-terminus which form the core of the complex together with the medium and small subunits [11]. Extending from each trunk via ~100 residue unstructured linkers are the COOH-terminal appendage globular domains [12,13]; these are predicted to be capable of extending from the core to sample the environment, but to spend most of their time in a retracted position [11,14,15]. The four subunits of F-COPI are predicted to associate in a complex resembling the AP2 complex. To date, the only known structure for coatomer is of the γCOP appendage, and although the sequence identity between γCOP and the large AP2 subunits is very low, the structural homology is striking [16,17] and together with consideration of the overall architecture of the complex, hints at a similar appendage in βCOP.

The adaptin-family appendages can be divided into two subdomains, an amino-terminal β-sandwich subdomain with a fold resembling that of immunoglobulins, and a COOH-terminal "platform" subdomain. The platforms of the γCOP and AP2 appendages each contain an FxxxW motif; in AP2 this motif has been demonstrated to mediate binding to a number of ligands important in the regulation of clathrin coat formation (reviewed in [18-20]). A second ligand binding site is present in the β-sandwich subdomain of both AP2 appendages [12,14,21,22]. Other adaptins and related proteins contain similar appendages with ligand binding functions (reviewed in [4]).

COPI vesicle formation is controlled by the small GTPase Arf1p which, in turn, is regulated by guanine nucleotide exchange factors (GEFs) and GTPase activating proteins (GAPs) (reviewed in [23-28]). The ArfGEFs belong to the Sec7 family of proteins (reviewed in [29]) and catalyze the exchange of GDP for GTP by Arf1p, causing the activation and membrane recruitment of Arf1p. ArfGAPs have a complex function that is currently a source of active discussion (reviewed in [30,31]). The hydrolysis of GTP by Arf1p is a prerequisite for vesicle uncoating and requires an ArfGAP as Arf1p has no intrinsic hydrolytic activity [32]. ArfGAPs also play roles in cargo packaging and the detection of membrane curvature, in addition to being a component of the vesicle coat [33-36]. There are six identified proteins containing ArfGAP domains in yeast: Age1p, Age2p, Gcs1p, Glo3p, Gts1p, and Sps18p. Of these, the first four have been demonstrated to have ArfGAP activity on Arf1p [37-40]. There is some redundancy among the yeast ArfGAPs, with Glo3p and Gcs1p having overlapping function in retrograde trafficking and forming an essential pair [38,40].

In this study we sought to examine if βCOP contained an adaptin-family appendage and if such a domain would be critical for COPI-coated vesicle formation. Using a combination of structural modeling, genetic, biochemical, and cell biological approaches, we provide supporting evidence that βCOP does indeed have an adaptin-family appendage and demonstrate that this domain is essential for βCOP function. We also identified the ArfGAP Glo3p as a downstream effector of Sec26p. This role of Glo3p is not dependent on its catalytic GAP activity, suggesting that this protein serves distinct functions in COPI vesicle trafficking that are independent from its regulation of the nucleotide bound state of Arf1p.

## Results

### An adaptin-family appendage domain is predicted for Sec26p

**Figure 1:**
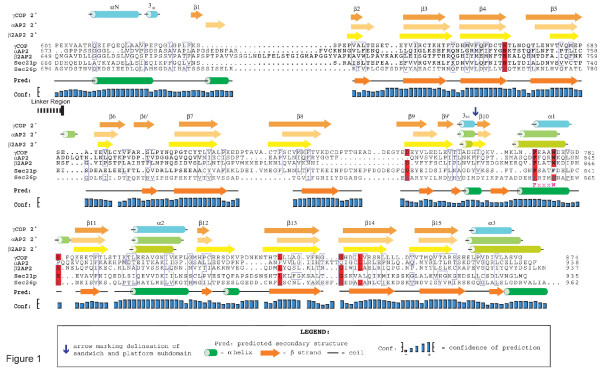
**Alignment of COPI and AP2 appendage domains**. A structure-based sequence alignment of the appendage domains of α- and βAP2 with that of γCOP was generated as described in [16]. Secondary structural elements of the γCOP and the AP2 appendage domains determined previously are indicated above the sequence alignment. The blue arrow denotes the position of the boundary between the platform and the Ig-like (β-sandwich) subdomains. Highlighted in red is where four or more sequences contained a strictly conserved residue at that position. Regions of similarity, calculated using functional amino acid groupings, are boxed in blue. Residues for which no structural information is available were positioned using a primary sequence alignment using the ClustalX 1.83 program and manual inspection. The conserved FxxxW motif is indicated below the sequence alignment in pink. The predicted secondary structural elements for the appendage domain of βCOP, Sec26p are shown beneath the alignment as determined with the PSIPRED confidence level of the assignments shown in the diagram.

Based on the known similarities in size, sequence, and structure of members of COPI to the AP2/clathrin coat, we predicted the Sec26p COOH-terminus to contain an adaptin-family like appendage domain [16]. To evaluate this possibility, we examined the sequence of Sec26p using two protein structure prediction servers, PSIPRED and mGenTHREADER [41-45]. Using PSIPRED, we found that the NH_2_-terminal 730 residues are predicted to be primarily α-helical in nature with some unstructured content (data not shown). The accuracy rate (Q_3_) of PSIPRED is considered to be approximately 78% per residue [46,47]. From residue 730 to 840, the protein is predicted to be composed of β-strands, and from residue 840 to the end (973), there is mixed content predicted. This overall domain architecture is similar to α- and β2AP2 which are composed of an NH_2_-terminal helical trunk domain [11], followed by a ~100 residue flexible linker, then an appendage domain which is composed of a β-sandwich subdomain (residues 701–824 in α, 705–825 in β2) and a platform subdomain (residues 825–938 in α, 826–937 in β2) [12,13,48]. Comparison of the secondary structure prediction for Sec26p to the known structures of the large AP2 subunits appendage domains suggests that Sec26p does indeed possess a similar COOH-terminal domain. Specifically, the β-sandwich domain is formed from residues 730–840, and the platform domain from residues 841–973. The boundary in Sec26p between the trunk domain and the predicted flexible linker region is not clear based on this prediction, but we expect that the trunk terminates at residue number ~600 as with the AP2 subunits. Whether there is a flexible linker connecting the two domains or if this region forms a distinct domain in Sec26p is unclear from the prediction. The output from the PSIPRED secondary structure server is illustrated graphically in Figure 1 together with an alignment of equivalent regions of β2AP2, αAP2 and γCOP.

mGenTHREADER is a protein fold recognition server. Input of the entire Sec26p sequence returns several "Certain" hits, i.e. matches with a confidence level > 99%. Included in this list are both the AP2 and related AP1 cores, specifically the αAP2, β2AP2, and γAP1 chains. Input of the sequence for residues 700–973 (i.e., the predicted appendage region) returns a single "Certain" (p < 0.0001) hit with a net score of 0.889, namely, the αAP2 appendage, which has 11.7% primary sequence identity to the Sec26p appendage. The next closest match is the γCOP appendage with a "Medium" certainty level (p < 0.01), with a net score of 0.439 and a percent identity of 16.2%. Third on the list, with a "Guess" certainty level (p >/= 0.1), is the β2AP2 appendage with a net score of 0.317 and a sequence identity of 12.9%. The sequence relationship of the NH_2_-terminal trunk domain of Sec26p with the other adaptin-family trunk domains, has been previously noted [49,50]. These results emphasize the conservation of fold of the trunk domains and additionally suggest a less stringent but still significant preservation of the adaptin-appendage domain fold.

In addition to structural predictive methods, sequence alignments were also performed to predict the appendage domain in Sec26p. Alignments between βCOPs and γCOPs suggest the appendage of Sec26p to begin at approximately residue 667, whereas the alignment with αAP2s suggests a start residue of 729, closer to that predicted by the secondary structure predictions. The platform subdomain is predicted to start at residue 849 based on the alignments with γCOPs and at residue 845 based on alignments with αAP2s, both very close to the start site predicted by the secondary structural prediction models. The appendage domains include several residues which are conserved amongst appendage domains (color coded red in Figure 1) including the FxxxW motif of the platform domain.

In summary, both secondary structure and fold recognition predictors assign the Sec26p COOH-terminus to the adaptin-appendage family with a high level of confidence. The combination of predictive algorithms places the start of the platform subdomain at approximately residue 840. The start of the β-sandwich subdomain is less precisely predicted, somewhere between residue 667 and 730 in Sec26p.

### The Sec26p appendage, specifically the platform subdomain, is essential in S. cerevisiae

**Figure 2:**
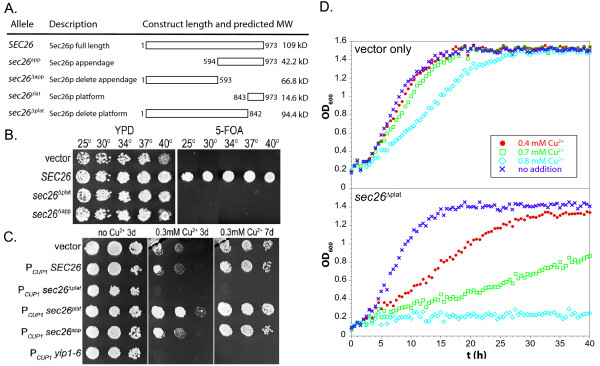
**Functional importance of the βCOP appendage domain**. A. Schematic illustrating the characteristics of the various Sec26p truncation constructs used for experiments. B. Plasmids coding for truncated constructs of *SEC26*, wild type *SEC26*, or vector only were transformed into the *sec26*Δ tester strain and spotted onto complete media (YPD) and synthetic complete media (SCD) with 5-FOA at 25, 30, 34, 37, and 40°C. C. Plasmids coding for vector alone or full length or truncated constructs under control of the copper-inducible promoter (P_*CUP1*_) were transformed into wild type yeast and replica-plated onto minimal media (SD) with and without the addition of Cu^2+ ^(0 or 0.3 mM CuSO_4_). Growth was followed for three or seven days (3d and 7d respectively). The dominant negative *yip1-6 *construct was included as a negative control [71]. D. Transformed cells were grown in liquid minimal media with and without the exogenous CuSO_4_. Cultures were grown to early log phase and adjusted to an OD_600 _of 0.2. CuSO_4 _was added at the levels indicated and cells were grown at 30°C with turbidity measured every half hour for 2 days.

To determine if the predicted Sec26p appendage domain was a critical functional unit, a truncated construct of *SEC26 *(973 residues total) coding for the trunk region alone was created (*sec26*^Δ*app*^; pRC2482, this study, see Figure 2A for construct schematic). This plasmid was transformed into the *sec26Δ *tester strain and plated on 5-FOA for plasmid shuffling. The resultant strain was inviable (Figure 2B), demonstrating the essential nature of the Sec26p appendage domain. A more conservative truncation (*sec26*^Δ*plat*^; pRC2521, this study) of the predicted platform subdomain also resulted in inviability (Figure 2B), demonstrating the critical nature of the function of the platform domain of the βCOP appendage. This is in contrast to deletion of the Sec21p appendage (*sec21*^Δ*app *^containing amino acids 1–676) region which is non-lethal [16].

### Over-expression of a truncated Sec26p missing the platform subdomain is dominant negative

The lethality caused by removal of the βCOP appendage or platform domain could be a result of the appendage/platform domain's critical function; however it was also possible that the truncations render the protein misfolded and subject to degradation, effectively the same as a deletion of the entire gene. Since the cell cannot function with *sec26*^Δ*plat *^at endogenous levels as the only copy of *SEC26*, we wished to test if this construct could provide function when over-expressed. The truncated gene was placed under control of a copper-inducible promoter (P_*CUP1*_) for over-expression (pRC2631a, this study). The truncated constructs (see schematic in Figure 2A) were transformed into wild type cells, and screened on solid minimal media containing approximately 0.3 mM CuSO_4_. As depicted in Figure 2C, over-expression of *sec26*^Δ*plat *^resulted in inviability on solid media. Growth curves in liquid media with varying amounts of CuSO_4 _(0, 0.4, 0.7, and 0.8 mM) reveal the dose-dependent nature of the dominant negative effect (Figure 2D). Over-expression of full length Sec26p, the appendage domain (residues 594–973; pRC2629a, this study) alone, or the platform subdomain (residues 843–973; pRC2632a, this study) alone, did not result in any detectable phenotype (Figure 2C). These results suggest that the appendage domain regulates the activity of Sec26p in the context of the holocomplex. The AP2 appendages are known to bind both unique and overlapping sets of partners, however, over-expression of the platform or appendage domain of Sec26p alone is not detrimental as might be expected if these appendage domains were to associate with binding partners and prevent their action on the holocomplex. This may be due to the fact that these interactions are of low affinity as suggested by the equivalent protein-protein interactions of the adaptin appendage domains [12-14].

### Random mutagenesis of Sec26p appendage domain reveals residues critical for function

The phenotype difference between Sec26p and Sec21p appendage domain deletion highlighted the essential nature of this region for COPI function. We created point mutations of the Sec26p appendage domain to further dissect the action of this domain at a molecular level. Initially, targeted mutagenesis was performed on a subset of charged residues chosen based on sequence alignments of fungal species. Eight residues were mutated to alanine residues, singly or in pairs as listed: K667 and K669, N751, D823, E828 and D829, and D903 and D904. Silent restriction enzyme sites were incorporated to facilitate the identification of correctly mutated constructs. Constructs were transformed into the *sec26Δ *tester strain and plated at 25 and 40°C on 5-FOA media to assess functionality of the mutant allele. None of the mutations resulted in a detectable phenotype (data not shown). A random PCR-derived error resulted in the trunk domain mutation *sec26-1 *(T536A D903A D904A; pRC2653a, this study). This construct was temperature sensitive, whereas other constructs containing the D903A D904A mutations were robustly wild type, suggesting that the phenotype was attributable to the T536A mutation.

**Figure 3:**
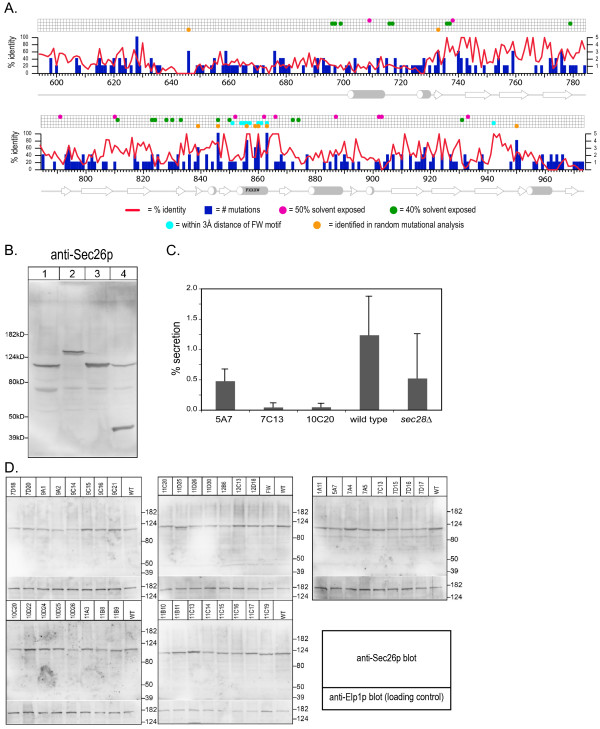
**Sec26p mutant allele creation**. A. Bar chart showing the relative location and number of each mutated amino acid position identified in the ts allele set created by random mutagenesis. Superimposed is the % identity of each residue derived from a sequence alignment of βCOP sequences in Clustal v1.83. For reference, a grid indicates the positions of amino acid residues that are aligned (according to Figure 1) with solvent exposed residues of γCOP, located within 3Å distance of the FxxxW motif, and identified as a minimal set of functionally important residues through *GLO3 *interactions. B. Expression of constructs revealed with anti-Sec26p appendage domain antibody. Whole cell lysates were prepared from (Lane 1) wild type cells expressing endogenous Sec26p (109 kDa), (Lane 2) cells expressing GFP-Sec26p as the only copy of Sec26p (136 kDa, pRC2798), (Lane 3) cells over-expressing Sec26p under control of the copper-inducible promoter (P_*CUP1*_) (42.2 kDa, pRC2630a), or (Lane 4) cells expressing both wild type levels of Sec26p plus over-expressed Sec26p appendage under control of the copper-inducible promoter (P_*CUP1*_) (pRC2629b). C. Invertase secretion in *sec26*^*ts *^mutant cells. Several mutant alleles were chosen and invertase secretion was measured after a one hour shift to the restrictive temperature. The graph shows % secretion relative to wild type and an alternative COPI ts mutant, the deletion of *sec28 *(εCOP subunit). Data plotted is the average of three experiments and confidence intervals shown with error bars. D. Sec26p allele expression levels. Cellular lysates prepared from each of the *sec26*^*ts *^strains after incubation at 40°C for 1 h to assess Sec26p expression levels at the restrictive temperature. Blots were subsequently probed with an anti-Elp1p (~150 kDa) antibody [70] as a loading control.

We next performed random mutagenesis on the Sec26p appendage to generate conditional lethal thermosensitive (ts) mutants. Error-prone PCR was performed on the appendage domain of Sec26p, leaving the trunk domain wild type. Tester strain transformants were screened for functionality after plasmid shuffling on 5-FOA plates incubated at 30 and 37°C. Thirty-eight unique ts mutants were generated from approximately 6000 transformants (Table 1) and contain between three and twelve point mutations each; two also contain 13-residue deletions at the COOH-terminus, and one contains an additional five residues at the COOH-terminus due to frameshift mutations. The temperature sensitivity profile of each mutant is shown in Table 1 and ranged from 34 to 40°C. In aggregate 276 mutations were identified, which occurred at 181 positions out of a total of 380 potential positions. Notably, five of these randomly generated mutants contained mutations in the FxxxW motif and there was a significant cluster of mutations identified that are close to this motif. Many of the mutated residues were represented two and occasionally three times. The number of mutations found occurring at each position is illustrated graphically in Figure 3A together with the position conservation and solvent accessibility data for residues that could be mapped onto equivalent positions in the γCOP structure.

**Table 1:** Summary of *sec26 *mutants and genetic interactions with *GLO3*

**Name**	**Mutations**	**Restrictive temperature^1^**	***GLO3 *suppression?**	***glo3*Δ synthetic lethality?**
**FW**	F856A W860A	40°	YES	YES
**TDD**	T536A D903A D904A (*sec26-1*)	34°	YES	YES
**7A4**	K659R I681V D759V S810P C905S I938V L950S	37°	YES	YES
**7D15**	S725Y K732N N772I H774R F777Y F799I K915M K964T	37°	YES	YES
**9C15**	C678S F693S D759G Y823C V838A I842T Y913H	37°	YES	YES
**10D22**	R672G I727T V743G K807R T877A	37°	YES	YES
**10D25**	L651Q K659E H839P I846T F863I A959S	37–40°	YES	YES
**11A3**	K659E K679N I733L Y744C F754S Y832N A851V F863L I893T I938L V973A	37°	YES	YES
**12B6**	N680T R691W D741G F817Y M859V	37–40°	YES	YES
**1A11**	F856A W860A D904A C905S	37–40	YES	+/-
**11D26**	E633D I733T D741V T769P F802L (*sec26-2*)	37°	YES	+/-
**12D18**	Q661R S740P V806A F907Y F918S	37°	YES	+/-
**11C15**	S628P I785V M859I L950S D956V H972Y	37°	YES	+/-
**7D20**	S740P I785T N793S K801E F863S A951T	37°	YES	no
**9A1**	I598N S728T D759V D823G I846T H855L I869L G901R	37°	YES	no
**10D24**	V623A S628T P636A V757A T779S W860R K887R T895A K933E A962T	37°	YES	no
**11B10**	L773Q I795T H839L N966Y	37°	YES	no
**11C13**	Q607L M624V L646P H670P C678S I846T T877I N890S E920V K963E	34°	YES	no
**9C16**	S626P K655R N793H T803S G818S N836D K868R E929K Q948L K963R	37–40°	YES	no
**5A7**	E633G N635Y E663G K666R F690L R691W S728L V756D Q776R D921G S932T L950M	34°	no	YES
**7C13**	F650L G695S H797Q I821N W860L E902G	34°	no	YES
**7D17**	K655N A665T D676G Q702R Q776H G781C Y822N D844E F863Y R883S	37°	no	YES
**7D18**	K614R L780H V794E A825V L885M C910R C927S A962S	37°	no	YES
**9A2**	Q607R K669M I846F M859V F907L F918Y	40°	no	YES
**9C14**	D697N D704H S740T I820T R831C I842V I928F S932T	37°	no	YES
**10C20**	S608T D616E D676G F693L D704G S726G P735H E746K H774Y F799L K847I F856Y	34°	no	YES
**10D26**	I598T R622G A718P T722I P735L K767E H839P T850S N890S D921E N966Y T971A	37°	no	YES
**11B11**	I615F L646Q K715R F817S F863L K872E M891L L950S	34–37°	no	YES
**11C14**	R622G K679G A694D A924V C927S V937D	40°	no	YES
**11D30**	A649E C910R S917P	37°	no	YES
**11C20**	D676N L730R F754L N772Y A830D I834N D852V S917T F918I	37°	no	YES
**7A5**	S619F L620V D709E R957G	40°	no	+/-
**11C19**	V600D F658S L712Q Q753H Q776R N836I T965A	37°	no	+/-
**7D16**	S628T L712Q P789S V794E D829V I846F I961F^2^	34–37°	no	no
**11B8**	I615F R660G S724C S916P K964E L970H	40°	no	no
**11B9**	E617V I629S K715I T889S A924V A959H^3^	34°	no	no
**11C16**	I662F V701G H720P V743G K784R F856I K887E	40°	no	no
**11C17**	L620I M624I L646P T654S T686M I749V V794G N867S K868G	37°	no	no
**11D25**	S628P A718S I733M P796T A825V Y832F^3^	37–40°	no	no
**12C13**	S628P G798C F802S E866G Y913C	34°	no	no

^1 ^restrictive temperature on SCGalactose media

^2 ^also contains additional residues at COOH-terminus ^974^WTTLS^978^

^3 ^also contains deletion of COOH-terminal residues 960–973

Because each mutant allele contained an average of 4.8 individual mutations that could potentially be destabilizing, we wished to determine if the temperature sensitive phenotypes of the mutant alleles of *SEC26 *were due to loss of expression at the restrictive temperatures. Whole cell lysates were analyzed by Western blotting using an antibody generated against a recombinant fusion protein of the Sec26p appendage domain (Figure 3B). The mutant strains were incubated at 40°C for one hour prior to lysis, a time period sufficient to observe a block in secretion measured by the accumulation of intracellular invertase (Figure 3C). Although some variation in expression is apparent in comparison to the loading control, none of the mutant strains exhibited significantly diminished protein levels or evidence of degradation after shift to the restrictive temperature, compared to wild type cells also incubated at 40°C (Figure 3D). These results suggest that the conditional temperature sensitive phenotypes are not the result of instability and altered Sec26p levels but are due to functional impairment of the Sec26p appendage domain.

### The ArfGAP Glo3p genetically interacts with sec26^ts ^mutants

One mutant allele identified through random screening, *sec26-2 *(11D26; pRC2948, this study), was found to contain 5 mutations restricted to the predicted β-sandwich subdomain (E633D I733T D781V T768P F802L), with no mutations in the platform subdomain. Because both of the appendages of AP2 have been identified to have two binding sites [12,14,21,22], one on the platform and one on the β-sandwich subdomain, and because related appendages without platforms (e.g., γAP1) also bind ligands [51], we selected this mutant to work with further, as a useful complement to the platform subdomain mutant, *sec26*^*FW*^.

**Figure 4:**
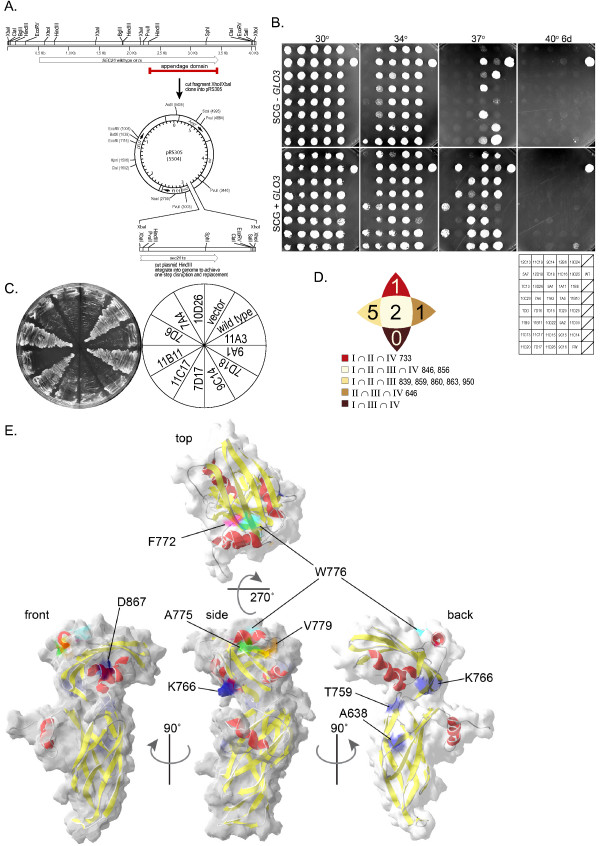
**Phenotype of *sec26*^*ts *^Mutants**. A. Schematic of strategy to create appendage domain mutants for suppressor screening. ts alleles created as described in Materials and Methods were subcloned into the vector pRS305 for one-step gene disruption and replacement to create integrated genomic *sec26 *alleles. B. The *sec26Δ *tester strain was transformed with each of the *sec26*^*ts *^mutant plasmids and plated on synthetic complete media (SCD) with 5-FOA to counter-select for the wild type *SEC26 *plasmid (not shown). The resultant strains were plated at 30, 34, 37, and 40°C to demonstrate the temperature sensitive phenotype of each mutant (top row). Each strain was transformed with a *GLO3 *expressing plasmid under control of the galactose-inducible promoter (P_*GAL1/10*_) and plated on SCG at the above listed temperatures to evaluate the ability of *GLO3 *to suppress the ts phenotype (bottom row). The data is also summarized in Table 1. C. The *sec26Δglo3Δ *tester strain was transformed with each of the *sec26*^*ts *^mutant plasmids and streaked onto SCD + 5-FOA to assess growth when *sec26*^*ts *^was expressed as the only copy of *SEC26 *in the *glo3Δ *background. One representative plate is shown. Data for all *sec26 *alleles is summarized in Table 1. D. Diagram showing the numbers of residues identified at the intersection of at least three of the Groups I-IV together with the residue number in Sec26p. E. Mapping of residues identified in Figure 4D onto the known structure of γCOP appendage domain. The γCOP appendage structure is depicted as a ribbon diagram with α-helices in red and β-strands in yellow. The calculated molecular surface (transparent grey) is overlaid on the ribbon diagrams. Selected residues (K766, T759, A638, D867) were identified from the alignment shown in Figure 1 as the equivalent of the Sec26p residues chosen as described in Figure 4D and shown in blue. For clarity, the residues surrounding and including the FxxxW motif are shown in additional colors: pink (F772), light blue (W776), green (A775), and orange (V779). The appendage is shown from a "side" view (center image) with the NH_2_-terminal α-helix projecting toward the viewer. The image was then rotated 90° for the "front" view (left image), 90° for the "back" view (right image), and 90° towards the viewer for the "top" view (top image). Each of the selected residues has surface exposure.

To seek candidate downstream effectors for the Sec26p appendage, we performed suppressor screens on two of the mutants to identify genetically interacting partners. The *sec26*^*FW *^and *sec26-2 *alleles were integrated into the genome as the sole copy of *SEC26 *for use in these screens to facilitate recovery of the suppressor plasmids (Figure 4A). These mutant strains were transformed with a galactose-inducible promoter (P_*GAL1/10*_) controlled over-expression library [52], and screened for thermoresistance at the restrictive temperature on galactose-containing media after an overnight recovery period at 30°C. Wild type *SEC26 *was recovered multiple times, and *GLO3 *was recovered twice from the *sec26-2 *screen. Suppression by *GLO3 *over-expression is partial and does not restore full wild type growth. The P_*GAL1/10 *_*GLO3 *plasmid (pRC2979, this study) was transformed into each of the appendage mutants and found to suppress a subset of them (Figure 4B, compare top and bottom row).

Since over-expression of *GLO3 *suppressed several of the *sec26*^*ts *^mutants, we examined the effect of *GLO3 *deletion in combination with the *sec26*^*ts *^mutants. Results are shown in Figure 4C. Most of the *sec26*^*ts *^mutant alleles were synthetically lethal with *glo3Δ*, and a few exhibited impaired growth. Evaluation of the pattern of suppression and synthetic lethality for *sec26*^*ts *^mutants with *GLO3 *revealed that one set of mutants were suppressed by *GLO3 *over-expression and synthetically lethal with *glo3Δ *(Group I), another two sets showed either suppression or synthetic lethality (Group II and III), and a fourth set did not show either type of genetic interaction (Group IV). We made use of these groups as additional functional information to identify the residues most significantly contributing to function amongst the 181 mutated positions resulting from the random mutagenesis screen. Only 9 residues were shared by at least three groups (Figure 4D). Using the structure and homology based alignment of Figure 1, we mapped each of these residues onto the γCOP appendage domain structure (Figure 4E). The equivalents of these residue positions in γCOP could be identified as a surface, solvent exposed amino acid on either the "back", "side" or "top" view of the domain, suggestive of a function that engages other proteins necessary to regulate or transduce COPI function.

### The relationship between Sec26p and Glo3p

Our genetic data clearly demonstrate an interaction between Sec26p and Glo3p. In order to determine if this was mediated by a direct physical interaction with the appendage, we performed in vitro co-precipitation assays with recombinant proteins. His_6_-tagged Sec26p appendage (His_6_-Sec26p^app^) was co-expressed with GST-tagged full-length Glo3p in *E. coli *however, we were unable to demonstrate the existence of a direct physical interaction between Glo3p and the Sec26p appendage in vitro (data not shown).

**Figure 5:**
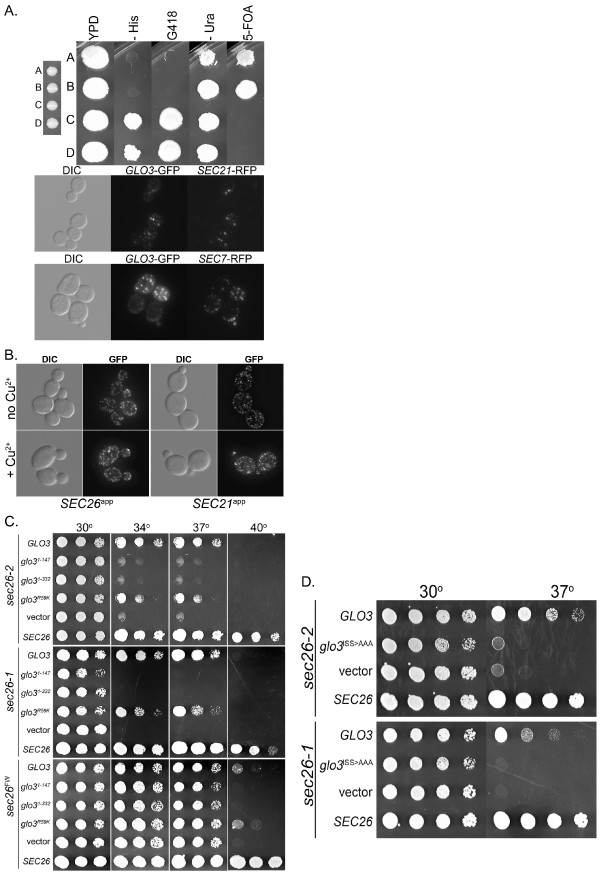
**Glo3p-GFP localization in the presence of over-expressed Sec26p appendage**. A. Functionality and localization of Glo3p-GFP. The functionality of the COOH-terminally tagged Glo3p-GFP construct (pRC3341A) was checked by transformation into the heterozygous diploid (*MAT***a**/α *his3Δ0/his3Δ0 leu2Δ0/leu2Δ0 ura3Δ0/ura3Δ0 MET15/met15Δ0 LYS2/lys2Δ0 GLO3/glo3Δ::HIS3 GCS1/gcs1Δ::*KAN^R^). Following sporulation, haploid strains deleted for the genomic copies of *GLO3 *and *GCS1*, were identified. Viable haploids that were His^+ ^and KAN^R ^were also positive for Glo3p-GFP and 5-FOA sensitive. Cells cotransformed with Glo3p-GFP and Sec21p-RFP or Sec7-RFP show coincidence of the green and red fluorescent signal in the punctate pattern typical of the Golgi of *S. cerevisiae*. B. Glo3p-GFP was expressed in a diploid *glo3Δ*/*glo3Δ gcs1Δgcs1Δ *background. This strain was transformed with a plasmid for over-expression of the Sec26p (residues E594-V973; pRC2629b) or Sec21p appendage (residues L676-Q935; pRC2628a) by a copper-inducible promoter (P_*CUP1*_). Cells were grown to early log phase, the cultures were divided, and treated with or without 0.7 mM CuSO_4 _for 3 hours at room temperature prior to microscopy. C. Selected *sec26*^*ts *^mutant strains (*sec26-1*, *sec26-2*, and *sec26*^*FW*^) were transformed with the *glo3 *truncation and point mutation constructs under control of P_*GAL1/10 *_and plated on SCG as a dilution series to evaluate the suppressive ability of the constructs. D. *GLO3 *suppression of *sec26*^*ts *^(*sec26-1 *and *sec26-2*) requires the ISS motifs located in the COOH-terminus of Glo3p. Mutant *sec26 *strains were transformed with the indicated constructs and tested for suppression as in 5C. The mutant ISS motif *glo3 *construct contained alanines at positions 388–390 and 420–422.

Since in vitro experiments did not reveal a direct physical interaction between Glo3p and the Sec26p appendage, we wished to determine if an interaction, direct or indirect, could be demonstrated in vivo. A previous immunofluorescence study in mammalian cells, showed that one of the Glo3p mammalian orthologs ARFGAP2, but not ARFGAP3, could be disturbed from its Golgi localization by over-expression of the mammalian γCOP appendage, and that this was dependent on an intact FxxxW motif [17]. To determine if Glo3p's intracellular localization was influenced by the βCOP appendage, we co-expressed Glo3p-GFP with over-expressed Sec26p appendage under control of P_*CUP1*_. This was performed in *glo3Δgcs1Δ *double knockout cells to eliminate any contribution from the functionally overlapping ArfGAP, Gcs1p. We first verified that Glo3p tagged with GFP at the COOH-terminus is a fully functional construct by testing its ability to act as the only retrograde Arf GAP in a double knockout of *glo3Δ *and *gcs1Δ*, a genetic background where *GLO3 *becomes essential for viability (Figure 5A). The appendage of Sec21p (γCOP) was also over-expressed to determine its effect on the localization of Glo3p. Results are shown in Figure 5B. We did not detect any difference in the localization of Glo3p-GFP in the presence of either appendage domain when over-expressed. Therefore, the localization of yeast Glo3p, unlike that of its ARF GAP ortholog ARFGAP2, is not dictated by the coatomer appendages and is dependent on additional, as yet unidentified factors and may be functionally be analogous to human ARFGAP3 [53].

### An additional function to the ArfGAP activity of Glo3p is suggested by genetic interactions with sec26^ts ^mutants

The GAP domain of Glo3p is found in the first third of the protein; little is known about the function of the remaining two thirds. Each known ArfGAP, including Glo3p, contains a Zn-finger motif in the GAP domain [37,54,55] that is essential for its Arf-interaction function. A conserved arginine is also part of this domain and is also required for catalytic activity [55-58]. In order to determine what domain or function of *GLO3 *was responsible for the suppression activity (e.g., if suppression is dependent on catalytic activity), we created truncation and point mutants of *GLO3 *under control of the regulatable promoter P_*GAL1/10*_. The two Glo3p truncation mutants: *glo3*^*1–147 *^and *glo3*^*1–332 *^respectively includes the ArfGAP domain only (pRC3297a, this study), and the first two thirds of the protein (pRC3298a, this study). We also created a full length construct of Glo3p with the catalytic arginine mutated to lysine (*glo3*^*R59K*^) under control of P_*GAL1/10 *_(pRC3299b, this study). Constructs encoding *GLO3 *and *glo3 *mutants were transformed into selected *sec26*^*ts *^strains and plated on galactose-containing media for over-expression. Suppression required a full length version of the protein, but did not require catalytic activity (Figure 5C). This pattern was observed for each of the three mutants tested (*sec26-1*, *sec26-2*, and *sec26*^*FW*^). These results suggest that Glo3p has a second function that resides in its COOH-terminal domain. The COOH-terminus of Glo3p contains two repeats of the amino acid sequence ISSxxxFG (residues 388–395 ISSDQLFG and residues 420–427 ISSSSYFG) which are critical for its ability to suppress the growth defect of *arf1-16 *[59]. We investigated the potential contribution of these motifs to the ability of Glo3p to suppress *sec26*^*ts *^(Figure 5D) with a *glo3 *388^AAA^390 420^AAA^422 construct. Together, results show that the COOH-terminal ISS motifs in Glo3p are necessary for the ability of *GLO3 *to suppress *sec26-1 *and *sec26-2 *mutant cells and that this activity is independent of the ability of Glo3p to stimulate GTP hydrolysis on Arf.

## Discussion

The appendage domain of the γCOP subunit of the COPI vesicle coat bears a striking structural resemblance to adaptin-family appendages despite limited primary sequence homology (12–16% identity). The adaptin appendages are integral to the regulation of clathrin-coated vesicles via FxxxW and other motifs. Both the γCOP appendage domain and equivalent region on βCOP contain the FxxxW motif; based on the conservation of this motif in βCOP, we predicted the existence of a functional appendage domain in βCOP (Sec26p in *S. cerevisiae*) [16]. We previously identified the thermosensitive allele *sec26*^*FW*^, which contains mutations of the conserved FxxxW motif (to AxxxA) in the platform subdomain [16] although mutation of the same motif in Sec21p (which is less tightly conserved as FxxxF) had no detectable effect. The 38 *sec26*^*ts *^appendage domain alleles identified in this study contained a total of 276 mutations distributed amongst 181 out of a possible 380 positions. Given that there were three to twelve point mutations in each allele, the set also probably contains "noise", mutations in residues that do not contribute to the phenotype, in addition to individual point mutations and groups of mutations that together contribute to the overall phenotype. To identify a core set of residues that reliably contributed to the phenotype, we considered the subset of residues that appeared to be frequently mutated and intersected in terms of a genetic interaction with *GLO3*. Although this is an arbitrary designation, we used this intersection of phenotypes to provide a stringent filter to select functionally important residues from the experimental set of 181 mutated positions. These 9 residues could be expected to be involved in either receiving or transmitting βCOP functionality given that these residues appeared analogous to surface exposed residues of γCOP that might be expected to engage in protein-protein interactions.

This collection of mutants represents the first set of conditional lethal *sec26 *alleles and will be a useful tool for dissecting Sec26p function in vivo and vitro as we gain more of a mechanistic and structural understanding of COPI function. The identification of a core set of 9 residues is undoubtedly a conservative underestimation of the mutated positions in the collection that contribute to Sec26p domain functions. With additional functional information about the appendage domain, we expect to be able to refine this analysis further and understand the mechanistic basis by which these residues contribute to appendage domain function.

In addition to shared or overlapping functions of the two appendage domains, the severity of effect in Sec26p appendage deletion or mutation suggests that its function contains at least one unique aspect that is more critical to cell physiology than for the equivalent domain in Sec21p. Similar actions have been proposed for the AP2 appendage domains suggesting a common mechanistic theme for these types of coat complexes [14,60]. Aside from the FW motif, analysis of the sequence and predicted structural alignments reveal that the residues used by adaptin domains to interact with ligands are chemically different in the case of β- and γCOP. These considerations highlight the fact that the Ig-like sandwich and the platform subdomain scaffolds provide enormous flexibility in the surfaces they offer for directed protein-protein interactions.

Does the predicted βCOP appendage have analogous function to the AP2 appendages, acting as a hub [60] to recruit protein partners essential to the regulation of COPI-coated vesicle formation? The mammalian γCOP appendage has been suggested to interact with Glo3p, an ArfGAP [17], and during the preparation of this manuscript, the βCOP appendage was demonstrated to interact with the calpain nCL-2 in stomach cells [61]. Interacting partners for the appendage domains may require the multiple low-specificity sites provided by oligomerization of the COPI coat on the two-dimensional surface of membranes, and may possibly be governed by regulatory modifications. Although we have not identified a direct binding partner for the βCOP appendage, and demonstrated that appendage domain over-expression was not dominant negative, our studies did reveal an interesting relationship between the βCOP appendage and the retrograde ArfGAP Glo3p. We demonstrate that impairment of Sec26p appendage domain function can be suppressed by Glo3p over-expression, independent of its catalytic activity, suggesting that the action of Glo3p is downstream from COPI.

Retrograde ArfGAPs such as Glo3p and mammalian ARFGAP2 and ARFGAP3 are known to bind to coatomer as demonstrated by both in vitro and in vivo assays and genetically interact with genes encoding coatomer subunits [17,58,62], however the specific region of Glo3p involved has not been clarified and the number of binding sites on coatomer also remains undefined. Mammalian ARFGAP1 has been shown to contain two coatomer binding sites, one in the catalytic domain, and one in the non-catalytic domain [63]. Additional studies to further explore the nature of the relationship between Glo3p, coatomer, membranes and cargo will be necessary to resolve these fundamental questions.

Our results do not address where coatomer and ArfGAPs interact physically in vivo, but do provide interesting insights into the multi-functional nature of Glo3p. What is most remarkable about this interaction is that *glo3*^R59K ^over-expression is able to suppress the *sec26*^*ts *^alleles to virtually the same extent as wild type *GLO3*. Cells expressing *glo3*^R59K ^as the only copy of *GLO3 *are inviable and display a variety of secretory defects including failure of vesicle budding, accumulation of ER membranes, impairment of CPY processing, and mislocalization of retrograde cargo, however the mutant protein is still able to bind coatomer in coprecipitation assays [58]. Over-expression of *glo3*^R59K ^in wild type cells does not impair growth (our unpublished data) and one attractive possibility is that Glo3p combines GAP and effector interactions within the same polypeptide. Taken together with our suppression data, this strongly suggests that Glo3p has critical functions in addition to its GAP activity and that this function involves the COOH-terminus of Glo3p operating in conjunction with Sec26p and COPI. Glo3p contains two repeats of the amino acid sequence ISSxxxFG. This motif is critical for its ability to suppress the growth defect of *arf1-16 *[59], and, as demonstrated in this study, for the ability of *GLO3 *to suppress *sec26*^*ts *^mutants. The mechanistic underpinnings of this motif are unclear at present; a search of the yeast proteome reveals the ISSxxxFG motif in other proteins of very divergent function (Ubr2p, Vba1p, Ssm4p, Ada2p, Fur1p, Fur4p and the HO endonuclease). Further studies are required to decipher how this motif functions and if it could play a more general role. It is interesting to note that this motif is conserved in the human orthologs of Glo3p ARFGAP2 and ARFGAP3 [53,59]. Outside the GAP domain, the homology between ArfGAP proteins varies, a divergence that reflects the fact that their mechanisms of localization and specific functions are also quite disparate. An important goal will be to establish paradigms to understand how the non-catalytic regions of ArfGAPs contribute to GAP activity and respond to the signal transduction pathways that transmit to, and act downstream of Arf proteins.

## Conclusion

In these studies we sought to determine if βCOP contains an adaptin-family appendage domain similar to that determined for γCOP. Sequence comparisons and structural prediction tools suggest that the fold of the COOH-terminus of Sec26p is strongly predicted to closely mimic that of adaptin-family appendages. Experimental approaches, using both deletion and mutagenic studies illustrate the critical role of the predicted Sec26p appendage domain and in particular the residues in close proximity to the FxxxW motif that are expected to form a platform-like subdomain. The essential nature of the appendage, the presence of a conserved motif (FxxxW) in the predicted platform subdomain, the weak but significant sequence homology with other appendages, and the predicted secondary structure and fold collectively support the existence of an appendage domain on βCOP that plays an important regulatory role in COPI function.

## Methods

### Yeast strains and media

Yeast strains were cultured using standard media and conditions [64]. Plasmid shuffling was carried out on synthetic complete media (SCD) with 2.5 mg/ml 5-fluoro-orotic acid (5-FOA). Yeast strains are listed in Table 2.

**Table 2:** *S. cerevisiae *strains used in this study

**Strain**	**Genotype**	**Source**
**RCY269**	*MAT***a ***ura3-52 leu2-3,112*	This lab
**RCY3130**	*MAT*α *his3Δ0 leu2Δ0 ura3Δ0 lys2Δ0 SEC26ΔKAN*^*R *^[pRS316 *SEC21 SEC26 *pRC2374]	This lab
**RCY3315**	*MAT***a ***ura3-52 leu2-3,112 SEC26Δ::sec26*^FW ^(F856A W860A)*LEU2*	This study
**RCY3661**	*MAT***a ***ura3-52 leu2-3,112 SEC26Δ::sec26*^ts ^11D26 (*sec26-2*, E633D I733T T768P D781V F802L) *LEU2*	This study
**RCY3674**	*MAT*α *glo3Δ::KAN*^*R *^*his3Δ0 leu2Δ0 ura3Δ0 lys2Δ0*	ResGen
**RCY3675**	MAT**a**/α *glo3Δ::KAN*^*R *^*his3Δ0/his3Δ0 leu2Δ0/leu2Δ0 ura3Δ0/ura3Δ0 LYS2/lys2Δ0 MET15/met15Δ0*	ResGen
**RCY3676**	*MAT*α *gcs1Δ::KAN*^*R*^*his3Δ0 leu2Δ0 ura3Δ0 lys2Δ0*	ResGen
**RCY3677**	*MAT***a**/α *gcs1Δ::KAN*^*R *^*his3Δ0/his3Δ0 leu2Δ0/leu2Δ0 ura3Δ0/ura3Δ0 LYS2/lys2Δ0 MET15/met15Δ0*	ResGen
**RCY3782**	*MAT*α *glo3Δ::HIS3 his3Δ0 leu2Δ0 ura3Δ0 lys2Δ0*	This study
**RCY3784**	*MAT***a ***glo3Δ::KAN*^*R *^*his3Δ0 leu2Δ0 ura3Δ0 lys2Δ0 met15Δ0*	This study
**RCY3785**	*MAT***a ***gcs1Δ::KAN*^*R *^*his3Δ0 leu2Δ0 ura3Δ0 lys2Δ0 met15Δ0*	This study
**RCY3786**	*MAT***a ***glo3Δ::KAN*^*R *^*his3Δ0 leu2Δ0 ura3Δ0 met15Δ0*	This study
**RCY3787**	*MAT***a ***gcs1Δ::KAN*^*R *^*his3Δ0 leu2Δ0 ura3Δ0 met15Δ0*	This study
**RCY3881**	*MAT***a ***glo3Δ::HIS3 his3Δ0 leu2Δ0 ura3Δ0 met15Δ0*	This study
**RCY3882**	*MAT***a ***gcs1Δ::HIS3 his3Δ0 leu2Δ0 ura3Δ0 met15Δ0*	This study
**RCY4038**	*MAT*α *his3Δ0 leu2Δ0 ura3Δ0 lys2Δ0 SEC26Δ::KAN*^*R*^*glo3Δ::HIS3 *[pRS316 *SEC21 SEC26*]	This study
**RCY4233**	*MAT***a ***his3Δ0 leu2Δ0 ura3Δ0 met15Δ0 gcs1Δ::KAN*^*R*^*glo3Δ::HIS3 *[pRS316 *GLO3*-GFP pRC3341A]	This study
**RCY4236**	*MAT*α *his3Δ0 leu2Δ0 ura3Δ0 lys2Δ0 gcs1Δ::KAN*^*R*^*glo3Δ::HIS3 *[pRS316 *GLO3*-GFP]	This study
**RCY4238**	*MAT***a**/α *his3Δ0/his3Δ0 leu2Δ0/leu2Δ0 ura3Δ0/ura3Δ0 LYS2/lys2Δ0 MET15/met15Δ0 gcs1Δ::KAN*^*R*^/*gcs1Δ::KAN*^*R *^*glo3Δ::HIS3*/*glo3Δ::HIS3 *[pRS316 *GLO3*-GFP]	This study

### Alignments and secondary structure predictions

The protein sequence for Sec26p was submitted to the PSIPRED v2.5 server [41,42,44] with "Mask Low Complexity Regions" option selected. This was repeated for GenTHREADER and mGenTHREADER [43,45]. For alignments, 17 βCOP (COPB_HUM, NP_057535.1, XP_508297.1, NP_001006467.1, NP_001002013.1, AAQ63171.1, COPB_DRO, XP_393132.1, NP_194877.2, BAC87706.1, P41810, CAH01736.1, CAB46767.1, CAF06042.1, CAB95500.1, CAD26416.1, AAT12307.1) and 20 γCOP sequences were downloaded from NCBI in FASTA format and loaded into CLUSTALX v1.83 [65]. Alignments were performed with the following penalties: for pairwise alignments, gap opening was set to 35, and gap extension 0.75; for multiple alignments, gap opening was set to 15, gap extension to 0.3. The delay was set to 30%. All βCOP sequences and all γCOP sequences were aligned separately in Multiple Alignment Mode, and the two resulting alignments were aligned in Profile Alignment Mode. The procedure was repeated for βCOP and 29 αAP2 sequences. The coordinates for the *B. taurus *γCOP appendage (PDB ID 1PZD) were downloaded from the RCSB Protein Data Bank [66] and loaded into DeepView/Swiss-Pdb Viewer v3.7 for image manipulation.

### Mutagenesis

All *SEC26 *truncations and mutants were created in pRS315 [67] for evaluation of functionality in the *sec26Δ *tester strain (RCY3130; *MAT*α *his3Δ0 leu2Δ0 ura3Δ0 lys2Δ0 SEC26ΔKAN*^*R *^[pRC2374; pRS316 *SEC21 SEC26*] [16]) after plasmid shuffling or dominant negative activity in a wild type strain (RCY239; *MAT***a ***ura3-52 leu2,3-112*). Non-essential *KAN*^*R *^single knockout strains were obtained from Research Genetics, Inc., Huntsville, AL; other knockouts were created using standard techniques.

Random mutagenic PCR was performed on the appendage domain of *SEC26 *using primers designed to anneal beginning at the coding sequence for residue E594 and ending 579bp downstream of the stop codon, and was carried out as described by Wilson and Keefe [68]. The mutated product was co-transformed into the *sec26Δ *tester strain with a linearized plasmid containing a gapped *SEC26 *gene (pRC2877, this study) for recombination to form the complete *SEC26 *ORF. Transformants were replica plated to two 5-FOA plates for plasmid shuffling and incubated at 30 and 37°C. Digital images of the plates were pseudocolored and overlaid to identify ts colonies. The plasmids were extracted and re-transformed into the tester strain to confirm thermosensitivity of the construct. Confirmed *sec26*^*ts *^alleles were sequenced at the Cornell Biotechnology Facility using Big Dye Terminator chemistry on an Automated 3730 DNA Analyzer (Applied Biosystems; Foster City, CA).

### Suppressor screens

To facilitate suppressor plasmid recovery during the screen, the *sec26*^*ts *^plasmids of interest were integrated into the genome. Plasmids were digested with XbaI and XhoI to obtain a 1.9kb fragment containing the mutant appendage, which was ligated into the integrating *LEU2 *vector pRS305. The resulting plasmid was linearized at a unique HindIII site located immediately upstream of the appendage start and transformed into yeast for a one-step gene disruption and replacement at the *SEC26 *locus. The integrated *sec26*^ts ^strains were transformed with a library under control of the inducible galactose promoter (P_*GAL1/10*_, kind gift of A. Bretscher) [52] and approximately 50,000 transformants screened per mutant to obtain complete coverage of the genome. Transformants were replica plated to galactose-containing synthetic drop-out media to induce gene expression and incubated at 30°C overnight before being shifted to restrictive temperature. Suppressor plasmids were isolated from thermoresistant colonies, re-transformed into the mutant strains to confirm suppressor activity, and screened by diagnostic restriction digest with HindIII to eliminate plasmids containing wild type *SEC26*. Novel suppressors were then sequenced as above and identified using the BLAST server at NIH.

### Microscopy

GFP was fused to the COOH-terminus of *GLO3 *following a short linker (GGPGG) and expressed under the control of its endogenous promoter in pRS316 (pRC3441a, this study). The haploid *gcs1Δ *::*KAN*^*R *^deletion strain was crossed to a *glo3Δ *::*HIS3 *strain transformed with the Glo3p-GFP plasmid to generate a double knockout *gcs1Δglo3Δ *strain containing Glo3p-GFP. Diploid double knockouts were generated by crossing complementary haploid double knockouts (RCY4238a, this study; *MAT***a**/α *his3Δ0/his3Δ0 leu2Δ0/leu2Δ0 ura3Δ0/ura3Δ0 LYS2/lys2Δ0 MET15/met15Δ0 gcsΔ *::*KAN*^*R*^*/gcs1Δ *::*KAN*^*R *^*glo3Δ *::*HIS3/glo3Δ *::*HIS3 *[pRC3341a]). Live cells were analyzed with a Nikon Eclipse E600 microscope equipped with a 100X (1.4NA) lens and 1X optivar (0.08 μm/pixel). A Sensicam EM High Performance camera (The Cooke Corporation; Romulus, MI) was used for image capture using IPLab 3.6.5 software (Scanalytics; Rockville, MD). Blind deconvolution was accomplished using AutoDeblur 9.3 software package (AutoQuant Imaging, Inc.; Watervliet, NY) for 25 ± 5 z stacks with a 0.2 μm slice size for 30 iterations.

### Preparation of anti-Sec26p appendage antibodies

GST fused to Sec26p residues E594-A862 in pGEX-4T-1 (pRC2908a, this study) was expressed in *E. coli *and purified from inclusion bodies. Post-sonication pellets were resuspended in Buffer A (50 mM Tris pH 7.9, 0.5 mM EDTA, 50 mM NaCl, 5% glycerol) with 2% DOC and washed twice with rocking for 15 minutes at room temperature in between. The purified inclusion bodies were resuspended in equal volume 2× sample buffer (4% SDS, 120 mM Tris pH 6.8, 20% sucrose, 5% β-mercaptoethanol, 0.005% bromphenol blue) and resolved by SDS-PAGE. The band containing the fusion protein was excised, cut into slices containing ~100 μg fusion protein, and stored at -20°C. Pre-laying hens were injected intramuscularly with ~100 μg purified fusion protein suspended in PBS every two weeks for a total of three injections. The first two injections were given with 1 part by volume Freund's adjuvant and 2 parts 2% Tween 80 in sterile phosphate-buffered saline (PBS). IgY antibody was partially purified from the egg yolks once laying commenced by emulsification in 25 ml PBS per egg brought to 100 ml with chloroform, followed by centrifugation to separate the aqueous layer. 0.002% sodium azide was added to the IgY-containing aqueous extract which was stored at 4°C.

### Invertase assay

2 OD_600 _units for each sample in duplicate were harvested from log phase cultures. One set of samples (t = 0) was washed, and resuspended in 1 ml 10 mM sodium azide and kept on ice. The other set was washed, resuspended in 1 ml YP + 0.1% D-glucose and incubated at 40°C with shaking for 1 hour (t = 1) and processed as for the t = 0 set. Each sample was processed for two measurements, external invertase was measured on whole cells and total invertase was measured after glass-bead lysis according to standard methods [69]. Percent secretion was calculated for each sample.

### Western blotting

For lysate preparation, cultures were grown to early log phase, 0.7 mM CuSO_4 _was added to the copper-inducible strains, and all cultures were incubated for 3 hours at 30°C to mid log phase (OD_600 _~0.5–0.8). 10 OD_600 _units of cells was collected, washed in 500 μl ice cold TAZ buffer (10 mM Tris pH 7.5, 10 mM NaN_3_), resuspended in 50 μl TAZ buffer plus protease inhibitors (4 mM PMSF, 10 μg/ml Pepstatin A, 2 mM benzamidine, 2 mM EDTA) and subjected to glass bead lysis for two minutes at 4°C. 70 μl 2× sample buffer was added and the samples heated to 60°C for 10 minutes prior to SDS-PAGE gel electrophoresis. The proteins were transferred to methanol pre-wetted 0.45 μm PVDF membrane (Immobilon-FL; Millipore, Billerica, MA) at 250 mA for 2 hours in transfer buffer (2 g/L Tris base, 14.4 g glycine/L) with 2% methanol. Membranes were probed with the chicken anti-Sec26p appendage antibody described above. The antibody was incubated with the membrane overnight at room temperature at 1:1000 in Tris-buffered saline plus 0.2% Tween-20 (TBST) with 5% nonfat milk. After three ten-minute washes in TBST, blots were probed with alkaline phosphatase conjugated goat anti-chicken antibody (Southern Biotechnology Inc.; Birmingham, AL) at 1:10,000 in TBST for 30 minutes, and then subjected to three additional washes in TBST. Membranes were washed in 100 mM Tris pH 9.5, 100 mM NaCl, 5 mM MgCl_2 _for 10 minutes and developed with phenylphosphate substituted 1,2 dioxetane (CDP-Star, Perkin-Elmer; Norwalk, CT) imaged in a Fujifilm LAS-3000 cabinet (Fuji Photo Film U.S.A., Inc.; Valhalla, NY).

To evaluate the expression levels of the Sec26p mutants, yeast expressing the *sec26*^*ts *^alleles as the only copy of *SEC26 *were grown to mid-log phase and then shifted to 40°C for 1 hour prior to lysis. The cultures were pelleted and resuspended at 10 OD_600 _units/ml in ice cold TAZ (10 mM Tris pH 7.5, 10 mM azide) buffer. 10 OD_600 _units were collected, and resuspended in 100 μl TAZ buffer plus protease inhibitors and subjected to glass bead lysis for two minutes at 4°C. An additional 150 μl lysis buffer plus protease inhibitors was added, and 300 μl 3× sample buffer (6% SDS, 180 mM Tris pH 6.8, 30% sucrose, 7.5% β-mercaptoethanol, 0.005% bromphenol blue) was added. Samples were heated at 80°C for 10 minutes and 10 μl resolved on an 8% 1.5 mm SDS-PAGE gel. Western blotting was carried out as described above, except the chicken anti-Sec26p appendage antibody was used at 1:2000. Blots were re-probed without stripping with an anti-Elp1 antibody [70] as a cell lysate loading control.

## List of abbreviations

The abbreviations used are: ER, endoplasmic reticulum; COPI, Coat Protein complex I; AP2, Adaptin Protein complex 2; GST, glutathione *S*-transferase; GFP, green fluorescent protein; Arf1, ADP-ribosylation factor-1; GEF, guanine nucleotide exchange factor; GAP, GTPase activating protein; ts, temperature sensitive; 5-FOA, 5-fluoroorotic acid; SCD, synthetic complete media; SCG, synthetic complete media with galactose as the sole carbon source; PBS, phosphate-buffered saline.

## Authors' contributions

CJD performed the experimental studies and together with RNC, was involved in the study design, analysis and interpretation of data, drafting the manuscript and figure preparation, PBR created several yeast strains and plasmids used and performed the initial experiments identifying the βCOP appendage domain as critical for viability, GRH and RAC made intellectual contributions to the conception of the study. All authors participated in the design of the study, critiqued and approved of the final manuscript.
